# Supplementary material for: Longitudinal relations among inattention, working memory, and academic achievement: testing mediation and the moderating role of gender
Source: PeerJ. 2015 May 19;3:e939. doi: 10.7717/peerj.939 (PMC4451022; doi:10.7717/peerj.939)
Supplement: Figure S1 [file peerj-03-939-s001.pdf]

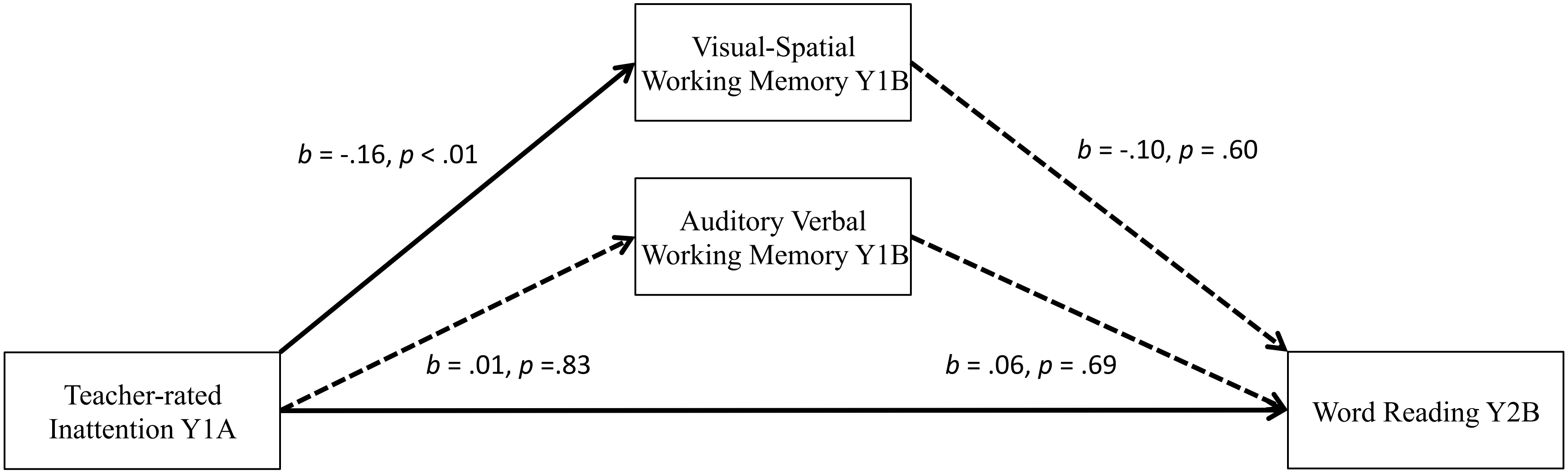

Direct effect, b = -0.02, CI [-0.10, 0.05]

Conditional indirect effects:
visual-spatial WM for boys, b = -0.00 CI [-0.02, 0.01]
visual-spatial WM for girls, b = -0.01 CI [-0.04, 0.02]
auditory-verbal WM for boys, b = 0.00 CI [-0.00, 0.02]
auditory-verbal WM for girls, b = 0.00 CI [-0.00, 0.01]
