# Supplementary material for: Longitudinal relations among inattention, working memory, and academic achievement: testing mediation and the moderating role of gender
Source: PeerJ. 2015 May 19;3:e939. doi: 10.7717/peerj.939 (PMC4451022; doi:10.7717/peerj.939)
Supplement: Supplemental Information 2 [file peerj-03-939-s002.docx]

| Supplemental Table 1  *Linear Model of Predictors of Visual-Spatial WM and Math Addition Fluency* | | | | |
| --- | --- | --- | --- | --- |
| Model | R^2^_adjusted_ | *b* | *SE B* | t p |
|  |  |  |  |  |
| Visual-spatial WM | 0.18** |  |  |  |
| Teacher-Rated Inattention |  | -0.16 | 0.06 | -2.92 *p* < .01 |
| Sex |  | 0.60 | 0.50 | 1.18 *p* = .24 |
| Sex * teacher-rated inattention |  | 0.07 | 0.04 | 1.94 *p* < .05 |
| Y1 Math Calculation |  | 0.04 | 0.02 | 1.98 *p* < .05 |
| Parent Education Level |  | -0.01 | 0.15 | -0.04 *p* = .96 |
| Age |  | -0.98 | 0.33 | -2.96 *p* < .05 |
| Year 2 Addition Fluency | 0.59** |  |  |  |
| Auditory-Verbal WM |  | 0.16 | 0.39 | 0.41 *p* = .68 |
| Visual-Spatial WM |  | 0.59 | 0.29 | 1.98 *p* < .05 |
| Teacher-Rated Inattention |  | -0.28 | 0.07 | -3.85 *p* < .001 |
| Y1 Math Calculation |  | 0.85 | 0.09 | 9.59 *p* < .001 |
| Parent Education Level |  | 0.53 | 0.59 | 0.88 *p* = .37 |
| Age |  | -1.31 | 1.37 | -0.95 *p* = .34 |
| ** *p* < .001 |  |  |  |  |
|  |  |  |  |  |
|  |  |  |  |  |
|  |  |  |  |  |
|  |  |  |  |  |
|  |  |  |  |  |
|  |  |  |  |  |
