# Supplementary material for: Longitudinal relations among inattention, working memory, and academic achievement: testing mediation and the moderating role of gender
Source: PeerJ. 2015 May 19;3:e939. doi: 10.7717/peerj.939 (PMC4451022; doi:10.7717/peerj.939)
Supplement: Supplemental Information 3 [file peerj-03-939-s003.docx]

| Supplemental Table 2  *Linear Model of Predictors of Visual-Spatial WM and Math Subtraction Fluency* | | | | |
| --- | --- | --- | --- | --- |
| Model | R^2^_adjusted_ | *b* | *SE B* | t p |
|  |  |  |  |  |
| Visual-spatial WM | 0.18** |  |  |  |
| Teacher-rated Inattention |  | -0.16 | 0.06 | -2.84 *p* < .01 |
| Sex |  | 0.68 | 0.51 | 1.33 *p* = .18 |
| Sex * teacher-rated inattention |  | 0.07 | 0.04 | 1.88 *p* = .06 |
| Y1 Math Calculation |  | 0.06 | 0.03 | 1.97 *p* < .05 |
| Parent Education Level |  | -0.01 | 0.15 | -0.05 *p* = .96 |
| Age |  | -0.97 | 0.33 | -2.95 *p* < .05 |
| Year 2 Subtraction Fluency | 0.55** |  |  |  |
| Auditory-Verbal WM |  | 0.59 | 0.30 | 1.98 *p* < .05 |
| Visual-Spatial WM |  | 0.52 | 0.23 | 2.25 *p* < .05 |
| Teacher-rated Inattention |  | -0.13 | 0.06 | -2.25 *p* < .05 |
| Y1 Math Calculation |  | 0.69 | 0.09 | 7.63 *p* < .001 |
| Parent Education Level |  | 0.79 | 0.46 | 1.70 *p* = .09 |
| Age |  | 2.02 | 1.04 | 1.95 *p* < .05 |
| ** *p* < .001 |  |  |  |  |
|  |  |  |  |  |
|  |  |  |  |  |
|  |  |  |  |  |
|  |  |  |  |  |
|  |  |  |  |  |
|  |  |  |  |  |
