# Supplementary material for: Longitudinal relations among inattention, working memory, and academic achievement: testing mediation and the moderating role of gender
Source: PeerJ. 2015 May 19;3:e939. doi: 10.7717/peerj.939 (PMC4451022; doi:10.7717/peerj.939)
Supplement: Supplemental Information 4 [file peerj-03-939-s004.docx]

| Supplemental Table 3  *Linear Model of Predictors of Visual-Spatial WM and Math Calculation* | | | | |
| --- | --- | --- | --- | --- |
| Model | R^2^_adjusted_ | *b* | *SE B* | t p |
|  |  |  |  |  |
| Visual-spatial WM | 0.19** |  |  |  |
| Teacher-Rated Inattention |  | -0.16 | 0.06 | -2.81 *p* < .01 |
| Sex |  | 0.64 | 0.50 | 1.28 *p* = .20 |
| Sex * teacher-rated inattention |  | 0.07 | 0.03 | 1.99 *p* < .05 |
| Y1 Math Calculation |  | 0.07 | 0.03 | 2.61 *p* < .01 |
| Parent Education Level |  | 0.00 | 0.15 | 0.01 *p* = .99 |
| Age |  | -0.09 | 0.31 | -0.28 *p* = .78 |
| Year 2 Math Calculation | 0.53** |  |  |  |
| Auditory-Verbal WM |  | 0.42 | 0.21 | 1.98 *p* < .05 |
| Visual-Spatial WM |  | 0.31 | 0.16 | 1.92 *p* = .05 |
| Teacher-Rated Inattention |  | -0.41 | 0.04 | -3.43 *p* < .001 |
| Y1 Math Calculation |  | 0.32 | 0.06 | 5.41 *p* < .001 |
| Parent Education Level |  | 0.45 | 0.33 | 1.36 *p* = .18 |
| Age |  | -2.22 | 0.68 | -3.29 *p* < .001 |
| ** *p* < .001 |  |  |  |  |
|  |  |  |  |  |
|  |  |  |  |  |
|  |  |  |  |  |
|  |  |  |  |  |
|  |  |  |  |  |
|  |  |  |  |  |
