# Supplementary material for: Longitudinal relations among inattention, working memory, and academic achievement: testing mediation and the moderating role of gender
Source: PeerJ. 2015 May 19;3:e939. doi: 10.7717/peerj.939 (PMC4451022; doi:10.7717/peerj.939)
Supplement: Figure S2 [file peerj-03-939-s005.pdf]

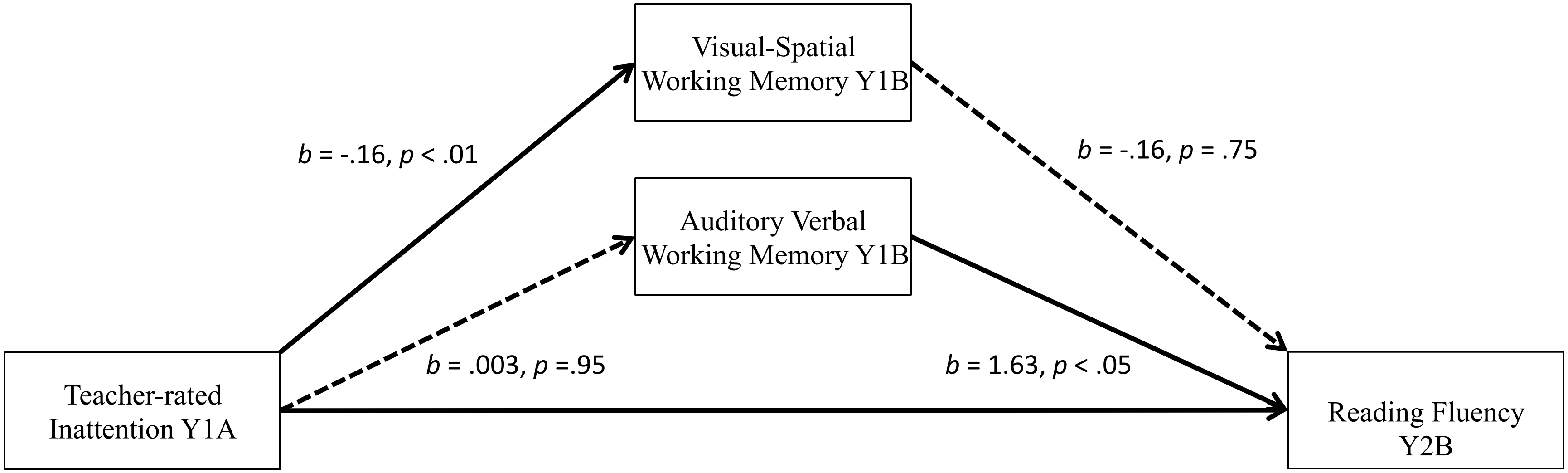


Direct effect, b = -0.27, CI [-0.54, 0.01]

Conditional indirect effects:
visual-spatial WM for boys, b = 0.00 CI [-0.02, 0.08]
visual-spatial WM for girls, b = 0.02 CI [-0.08, 0.15]
auditory-verbal WM for boys, b = -0.06 CI [-0.18, 0.00]
auditory-verbal WM for girls, b = -0.03 CI [-0.15, 0.02]
